# Pharmacological mechanism of natural products to treat osteoporosis: a focus on the autophagy

**DOI:** 10.3389/fphar.2025.1623990

**Published:** 2025-08-01

**Authors:** Chenglong Xin, Guangheng Zhang, Zheng Shen, Weibiao Han, Ruifeng Fan, Jiakuo Ren, Jianyi Zhang, Yanke Hao, Jian Xin

**Affiliations:** ^1^ The First Clinical Medical College, Shandong University of Traditional Chinese Medicine, Jinan, China; ^2^ School of Acupuncture and Massage, Shandong University of Traditional Chinese Medicine, Jinan, China; ^3^ Affiliated Hospital of Shandong University of Traditional Chinese Medicine, Jinan, China

**Keywords:** natural products, osteoporosis, autophagy, osteoclast, osteoblast

## Abstract

Osteoporosis (OP) is a serious public health issue, and fragility fractures resulting from OP are a major cause of death in the elderly. Osteoblast (OB)-mediated insufficient bone formation and osteoclast (OCs)mediated abnormalities in bone destruction can result in OP. Autophagy is the process by which cells degrade and recycle their own proteins and organelles. The differentiation and activity of OBs, OCs, and other bone cells depend on autophagy activity. The regulation of autophagy has the potential to influence the metabolic processes of these cells, which may contribute to the treatment of OP. This paper provided a comprehensive review of the experimental and clinical evidence supporting the use of natural products as potential therapeutic agents for OP. We examined the diverse regulatory effects of natural products on bone cells, including bone marrow mesenchymal stem cells, OBs, and OCs. Additionally, we explore the potential of these natural products to mediate autophagy, a process that may offer novel drug options and provide guidance for future clinical trials.

## 1 Introduction

Osteoporosis (OP) is the most common bone disease, characterized by the gradual loss of bone mass, low bone mineral density, and decreased bone strength, leading to fragile bones that are more susceptible to fractures (Roux, 2004). Bone metabolism is a complex process involving numerous cell types, including bone marrow mesenchymal stem cells (BMSCs), osteoblasts (OBs), osteoclasts (OCs), and osteocytes. These cells interact with each other to regulate the continuous remodeling of bone tissue (Florencio-Silva et al., 2015). BMSCs are located at the apex of OB differentiation. These cells can differentiate into OBs, chondrocytes, adipocytes, neuronal cells, and a variety of other tissue cells (Kang et al., 2000). OBs play an essential role in bone production and are crucial for bone maintenance (Capulli et al., 2014). OCs are the only cells capable of degrading bone tissue and are key factors influencing OP (Charles and Aliprantis, 2014). Bone cells, the final differentiated form of OBs, make up the majority of the mature bone cell population and play a key role in bone remodeling (Robling and Bonewald, 2020). It involves a series of physiological processes, including bone resorption, the recruitment of OBs and BMSCs, OC differentiation, and the completion of bone mineralization. The coordination of bone resorption and formation is essential for the maintenance of bone homeostasis, a phenomenon known as “coupling” (Martin and Seeman, 2007). Disruptions in any of these cell types can lead to OP (Šućur et al., 2014). OP has become a major public health challenge, and its incidence is expected to continue growing in the coming decades. Therefore, elucidating the molecular mechanisms involved in bone loss and developing novel therapeutic strategies is essential.

The fundamental pathogenesis of OP is characterized by a disruption of bone balance, manifesting as a disproportionate loss of bone mass relative to bone formation (Song et al., 2022). As research progresses, novel mechanisms underlying the pathological process of OP have been elucidated. For instance, the metabolites of gut microbiota have been demonstrated to exert substantial effects on bone metabolism, and the concept of the “gut-bone axis” is currently informing clinical practice (Zheng et al., 2025). In addition, Yes-associated protein (YAP)/PDZ binding motif transcriptional coactivator (TAZ) receives mechanical signals and converts them into biological signals. This finding indicates that targeting YAP/TAZ in mechanobiological signaling may be a viable therapeutic approach for OP (Chen et al., 2025). Among the many mechanisms, autophagy, a highly conserved catabolic process, is responsible for maintaining intracellular homeostasis by degrading damaged organelles and proteins and recycling their nutrients and energy. This process plays an important protective role in OP induced by oxidative stress environment (Yin et al., 2019; Li et al., 2024). In view of the discovery of these new mechanisms, natural products—compounds derived from natural sources such as plants, animals, and microorganisms—are regarded as promising sources and viable strategies for the development of novel OP therapeutic drugs. These natural products possess various pharmacological properties, including anti-inflammatory and anti-oxidant effects, as well as the capacity to induce fewer adverse reactions and to be more suitable for long-term use. Moreover, they demonstrate the potential to treat OP through multi-target mechanisms (An et al., 2016; Martiniakova et al., 2020; Yang et al., 2023). In light of the aforementioned background, the present review focuses on the research progress linking OP and autophagy, and systematically summarizes the natural products targeting the autophagy pathway for the treatment of OP. The objective of this review is to provide new ideas for the clinical treatment of OP.

## 2 Review methodology

**FIGURE 1:**
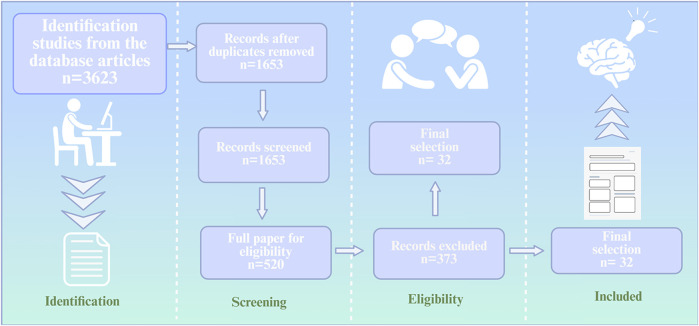
Flow diagram showing the inclusion and exclusion criteria used in the literature.

To investigate how natural products exert anti-osteoporosis effeets by regulating autophagy, we conducted a comprehensive search of several databases, including PubMed, Web of Seience, and ScienceDirect databasesbased on the PRisMA. The keywords used were “natural produets,” “autophagy,” “osteoblasts,” “osteoclasts,” “bone metabolic disorders,” “bone homeostasis,” “oxidative stress,” and “senility.” The retrieved articles were reviewed by two independent reviewers based on their title, abstract, and full text, adhering to inclusion and exclusion criteria, The inclusion criteria include: (1) original articles written in English; (2) articles set out to investigate the mechanisms by which natural products regulate autophagy in the treatment of osteoporosis in osteoporosis models. Exclusion criteria include: (1) articles written in any language other than English; (2) gray literature; (3) editorials; (4) review articles; (5) duplicate publications. Following the literature search and screening process, 32 articles were ultimately included in the evaluation (Figure 1).

## 3 Overview of autophagy

The term “autophagy” is derived from the ancient Greek word for “self-eating,” referring to the process by which a cell breaks down its own components (Yang and Klionsky, 2010). The types of autophagy currently reported include chaperone-mediated autophagy, microautophagy, and macroautophagy (Wang and Qin, 2013). In chaperone-mediated autophagy, proteins containing the KFERQ peptide sequence are recognized exclusively by the hsc70-containing protein complex. These proteins are then directed to the lysosome, where they are further recognized by the chaperone-mediated autophagy receptor, lysosome-associated membrane protein type 2A (LAMP-2A). The entry of LAMP-2A into the lysosome is facilitated by hsc70 (Orenstein and Cuervo, 2010; Arias and Cuervo, 2011; Penaloza et al., 2008). Microautophagy, on the other hand, involves the direct phagocytosis of cytoplasm by lysosomal membranes through invagination and entrapment. The turnover of peroxisomes in fungi under specific conditions is a typical example of microautophagy (Mijaljica et al., 2011). Macroautophagy, the focus of our present study and the most extensively studied form of autophagy, is characterized by the formation of double-membrane intermediate organelles known as autophagosomes. These vesicles contain various cytoplasmic structures, including soluble substances and organelles (Mizushima et al., 2011). Autophagy, a self-eating process observed in mammals, occurs under basal conditions and can be induced by various stimuli, such as starvation, oxidative stress, or rapamycin treatment (Wang and Qin, 2013). During starvation, autophagy conserves nutrients, breaks down cellular waste, generates energy, and produces new proteins and membranes, thus maintaining normal turnover of cytoplasmic components. Thus, autophagy is a metabolic process essential for maintaining cell mass and controlling energy homeostasis (Komatsu et al., 2005). Autophagy can be categorized into two distinct classifications: non-selective autophagy and selective autophagy. Non-selective autophagy is primarily triggered by starvation stimulation, while selective autophagy facilitates cell quality control by precisely identifying and degrading specific substrates, including damaged proteins, dysfunctional mitochondria, and protein aggregates (Reggiori et al., 2012; Wang and Qin, 2013). The primary function of selective autophagy is to differentiate between functional substrates and abnormal cellular components, thereby ensuring the maintenance of cellular homeostasis (Reggiori et al., 2012). In comparison with non-selective autophagy, selective autophagy has been demonstrated to play a more critical role in the regulation of cell mass balance (Chen and Klionsky, 2011; Isakson et al., 2013).

The process of autophagy is characterized by its high degree of conservation and includes several key steps: initiation, nucleation, elongation, maturation, and degradation (Yin et al., 2016; Markaki and Tavernarakis, 2020). In response to external stresses, the mammalian mechanistic target of rapamycin (mTOR)/AMP-activated protein kinase (AMPK) pathway induces the initiation of autophagy, or macroautophagy, through the Unc-51-like Kinase (ULK)- Autophagy-related protein (ATG) 13-Focal Adhesion Kinase Family Interacting Protein of 200 kDa (FIP200) complex. This complex is known for its remarkable stability and independence from nutritional status. Additionally, the formation of the ULK-ATG13-FIP200 complex is crucial for the binding of ATG101 to ATG13, a process essential for the initiation of autophagy (Jung et al., 2009; Hara et al., 2008; Ganley et al., 2009; Hosokawa et al., 2009a; Hosokawa et al., 2009b; Mercer et al., 2009). A complex composed of Vacuolar Protein Sorting (VPS) 34, VPS15, and BECN1 then binds to ATG14, engaging in the nucleation process (Liang et al., 1999; Kihara et al., 2001; Furuya et al., 2005; Itakura et al., 2008; Yan et al., 2009). Further control of phosphatidylinositol 3-phosphate (PI3P) synthesis promotes the elongation process of autophagy (Vergne and Deretic, 2010).

**FIGURE 2:**
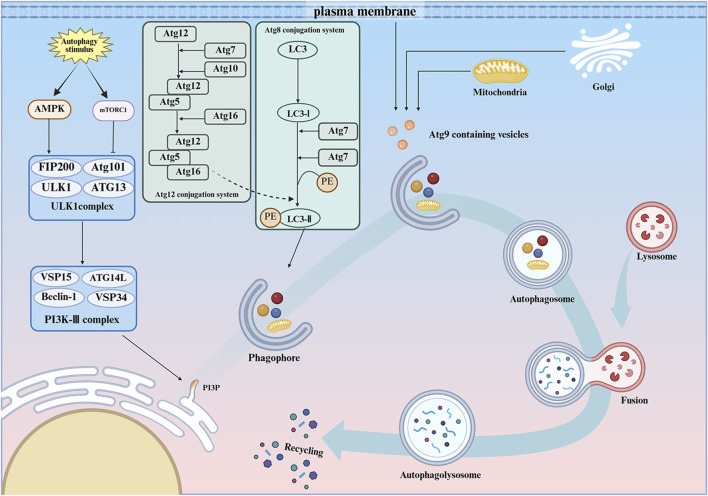
The ontogeny of autophagy. Autophagy is a process that maintains cellular mass and energy balance when a cell is exposed to external stresses, and is divided into different stages such as initiation, nucleation, extension, maturation, and degradation. AMPK, AMP-activated protein kinase; mTORC1, mechanistic target of rapamycin kinase complex 1; ULK1, Unc-51-like kinase 1; ATG13, Autophagy-related 13; FIP200, Focal adhesion kinase family interacting protein of200kD; ATG101, Autophagy-related 101; PI3K-Ⅲ, class III phosphatidylinositol 3-kinase complex; Beclin1, The mammalian yeast ortholog ATG6/VPS30; VPS34, Lipid kinase VPS-34 protein; VPS15, Lipid kinase VPS-15 protein; ATG14L, Autophagy-related gene 14; PI3P, Phosphatidylinositol 3-phosphate; ATG8, Autophagy-related 8; PE, Phosphatidylethanolamine; Microtubule-associated protein 1 light chain 3; ATG3, Autophagy-related 3; ATG7, Autophagy-related 7; ATG12, Autophagy-related 12; ATG10, Autophagy-related 10; ATG5,Autophagy-related 5; ATG16, Autophagy-related 16; ATG4,Autophagy-related 4; ATG9,Autophagy-related 9.

During the elongation of mammalian autophagy, two ubiquitin-like protein linkage systems contribute to phagosome expansion. The first system is the ATG12 system, which involves the formation of ATG12-ATG5-ATG16 (Popelka et al., 2021). The second system is the ATG8/light chain 3 (LC3) system, which involves the transformation of LC3 subfamily of microtubule-associated proteins (Sun et al., 2014). In the ATG12 system, the activation of ATG12 is initiated by ATG7, which binds to ATG5 in the presence of ATG10. This complex then interacts with ATG16, resulting in the formation of the ATG12-ATG5-ATG16 complex. This complex enhances the binding of LC3-I to phosphatidylethanolamine on phagosomes, mediated by ATG3 and ATG7. The complex is then converted to membrane-bound LC3-Ⅱ, which dissociates upon completion of the autophagosomes (Kraya et al., 2014; Wilson et al., 2014). Another protein identified as a potential contributor to autophagy is ATG9. Andrew et al. suggest that in mammals, ATG9 is located in the trans-Golgi network and late nuclear endosomes. When autophagy is upregulated ATG9 is redistributed to peripheral endosomal membranes, a process dependent on the activity of ULK1 and PI3K (Young et al., 2006). MAPK14/p38α has been shown to exert a negative regulatory effect on this process (Young et al., 2006; Webber and Tooze, 2010). During autophagy, enlarged phagosomes move toward and fuse with endosomes and/or lysosomes, ultimately forming an autophagic vacuole. The fusion of the phagosome with endosomes is facilitated by the protein VTI1B (Atlashkin et al., 2003), while fusion with lysosomes involves the SNARE complex, Rab7, and other proteins (Jäger et al., 2004; Fader et al., 2009; Furuta et al., 2010) (Figure 2).

## 4 The role of autophagy in OP

### 4.1 Clinical studies

The extant evidence for a potential association between aberrant autophagy function and osteoporosis is predominantly derived from *in vivo* experiments in animals and *in vitro* studies. The paucity of research in this area is evident, particularly in the context of human studies. Trojani et al. conducted an analysis of the bone tissue of patients with OP, and their findings indicated that the expression level of LC3-II was diminished, and the level of autophagy was reduced in the cortical bone of OP patients when compared with non-OP patients. This phenomenon may be attributable to the diminished expression of the hormonally regulated neural-associated kinase (HUNK) protein in patients with OP. HUNK protein has been shown to inhibit autophagy inhibitor Rubicon through a process of phosphorylation, thereby promoting autophagy. (Trojani et al., 2024). Tchetina et al. demonstrated significant downregulation of mTOR, RUNX2, and ALPL gene expression, significant upregulation of ULK1, p21, and MMP-9 gene expression, and upregulation of autophagy in the peripheral blood of postmenopausal women with OP. Furthermore, they showed that upregulated levels of autophagy increase osteoclast survival in the context of stable levels of expression of genes involved in osteoclast differentiation and activity, which in turn leads to increased bone loss. Consequently, this resulted in exacerbated bone loss and intensified the severity of the OP (Tchetina et al., 2015). In the study conducted by Yin et al., the therapeutic efficacy of the Zhuanggu Zhitong Recipe was investigated in patients suffering from OP who had entered the postmenopausal phase (Yin, 2024). Zhuanggu Zhitong Recipe, an effective Chinese medicine prescription, has been used for the prevention and treatment of OP. This prescription is composed of fructus psoraleae, epimedium, wolfberry fruit, etc (Ma et al., 2021). The findings of Yin et al. indicated that the Zhuanggu Zhitong Recipe could enhance the level of cellular autophagy by modulating the level of AMPK in patients suffering from OP. Additionally, it was observed that the Zhuanggu Zhitong Recipe could augment the bone mineral density of these patients, which suggests a potential role for this recipe in the treatment of OP (Yin, 2024). Furthermore, a study based on genome-wide association studies of wrist ultradistal radius (UD BMD) found that autophagy-related pathways were significantly correlated with UD BMD (Zhang L. et al., 2010). The findings of these studies lend support to the notion that autophagy plays a role in human OP.

The etiology of OP is multifactorial, but the underlying biological mechanism involves a disruption in the balance between bone formation and bone destruction, with the process tilting toward bone destruction (Yin et al., 2019). Bone tissue is subject to continuous remodeling, a process orchestrated by the concerted actions of various bone cells, including OCs, OBs, and bone lining cells. Dysregulation of any of these processes can result in bone loss, consequently leading to OP. Maintaining the dynamic equilibrium between OB-mediated bone formation and OC-mediated bone destruction is of paramount importance in OP (Crockett et al., 2011; Florencio-Silva et al., 2015). Autophagy is a highly conserved cellular self-catabolic process that is actively involved in the functions of all bone cells. Its initiation in oxidative stress environments can serve to protect bone cells, so it is inevitably associated with OP. Rubinsztein et al. suggested that aging, one of the key factors contributing to OP, leads to a decrease in autophagic activity, which is a direct cause of age-related OP (Rubinsztein et al., 2011). However, an analysis of the extant literature suggests that appropriate levels of autophagy activation in BMSCs and OBs play an important role in preventing and treating OP. Conversely, over-activation of autophagy in OCs can lead to OP.

### 4.2 Preclinical studies

#### 4.2.1 The role of autophagy in BMSCs

Autophagy has been demonstrated to play a pivotal role in the function of all OBs. Furthermore, the number and function of BMSCs, which serve as precursor cells to OBs, are crucial in the pathogenesis of OP (Qadir et al., 2020). The promotion of BMSC proliferation and their subsequent differentiation to OBs has been demonstrated to stimulate bone formation, thereby contributing to the alleviation of the OP process. The induction of autophagy is essential for the survival of BMSCs under oxidative stress conditions. Moreover, autophagy has been shown to maintain the stemness of BMSCs, a process that is regulated in coordination with AMPK/Akt/mTOR signaling, promoting the differentiation of BMSCs into OBs (Pantovic et al., 2013; Song et al., 2014; Hou, 2013). In contrast, inhibition of autophagy in BMSCs leads to the accumulation of reactive oxygen species (ROS) and DNA damage, which in turn triggers apoptosis in BMSCs (Hou, 2013). Ugland et al. demonstrated that autophagy activates BMSCs via the cAMP signaling pathway. Moreover, activated cAMP inhibits cell proliferation by regulating the activation of cell cycle protein E. This process recruits Beclin1 to encircle the nucleus and form autophagosomes (Ugland et al., 2011). The products of autophagosomal degradation can be oxidized by mitochondria, providing energy for differentiation (Oliver et al., 2012). However, the role of autophagy in mesenchymal stem cells (MSCs) remains largely unknown (Guan et al., 2013; Oliver et al., 2012). Li et al. investigated the application of rapamycin to induce autophagy in BMSCs, resulting in a decrease in the number of BMSCs in the S phase and an increase in apoptosis. In contrast, cells treated with 3-MA and chloroquine (CQ) exhibited the opposite effect (Li et al., 2016). This finding indicates that autophagy is a multifaceted process, exhibiting both beneficial and detrimental effects. Under conditions of significant stress, autophagy promotes cell survival. However, overactivation of autophagy can result in the degradation of proteins and organelles essential for cell proliferation, ultimately triggering apoptosis (Shintani and Klionsky, 2004). Consequently, moderate levels of autophagy activation have been shown to enhance the proliferation of BMSCs and induce their differentiation toward osteogenesis, thereby increasing bone formation and alleviating the OP process. However, excessive autophagy activation has been observed to result in the death of BMSCs and a decrease in their level of osteogenic differentiation, as well as an increase in their lipogenic differentiation. This, in turn, has been shown to induce or aggravate OP.

#### 4.2.2 The role of autophagy in OBs

OBs are responsible for the secretion of the organic matrix that constitutes bone tissue, as well as its mineralization. Additionally, these cells regulate the process of bone formation (Huitema and Vaandrager, 2007). Liu et al. specifically knocked down FIP200 in mouse OBs, resulting in impaired terminal differentiation of OBs and inhibition of bone formation, which led to OP. In contrast, post-treatment of BMSCs with 3-MA and CQ resulted in a decrease in the number of alkaline phosphatase (ALP)-positive cells. However, these treatments did not affect the early differentiation of OBs, suggesting that autophagy may be involved in the terminal differentiation process (Liu et al., 2013). Treatment with CQ did not appear to affect biomarkers for OB-related genes, indicating that the initiation of autophagy does not impair OB activity (Lin et al., 2016). The differentiation of BMSCs into OBs involves the inhibition of AMPK/mTOR activation, which in turn activates the Akt/mTOR signaling axis during both the early and late stages of autophagy (62). Negative pressure wound therapy, a subject of contemporary clinical trials, has been shown to promote OB differentiation and stimulate bone regeneration through the activation of AMPK, the induction of ULK1 phosphorylation, and the initiation of autophagy (Zhang et al., 2020). A similar effect is observed with insulin-like growth factor 1, which promotes OB differentiation and function by activating AMPK. Autophagy plays a direct role in the mineralization function of OBs. Deletion of the Beclin-1 or ATG7 genes has been shown to significantly reduce the mineralization capacity of OBs (Nollet et al., 2014). Similarly, specific knockdown of FIP200 or ATG5 in transgenic mice also resulted in impaired mineralization (Thomas, 2019). These results highlight the importance of autophagy in OB differentiation and mineralization. Moderate activation of OBs autophagy has been shown to increase the number of OBs, promote OBs function, and alleviate OP. However, overactivation of OBs autophagy can have the opposite effect.

#### 4.2.3 The role of autophagy in OCs

**FIGURE 3:**
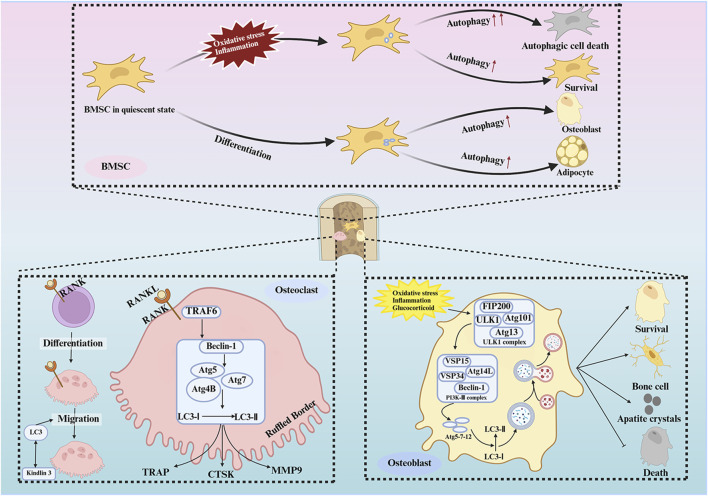
Autophagy of bone cells in osteoporosis. Autophagy in BMSCs is activated in response to external stimuli and during the differentiation of BMSCs, and the specialized differentiation of BMSCs is regulated by autophagy. Autophagy in osteoblasts is activated in response to a variety of stimuli to protect osteoblasts from apoptosis and to help osteoblasts adapt to harsh environments. In addition, autophagy influences osteoblast differentiation and mineralization. In addition, autophagy affects the differentiation and mineralization of osteoblasts. Osteoclasts need to maintain an appropriate level of autophagy for their differentiation, survival, migration and bone resorption.

OCs are large, multinucleated cells derived from hematopoietic mononuclear stem cells, and they play a crucial role in bone resorption. They are activated by two key factors: nuclear factor-κB ligand receptor activator (RANKL) and macrophage colony-stimulating factor (M-CSF), both of which are essential for osteoclastogenesis (Teitelbaum, 2000; Xu and Teitelbaum, 2013; Shapiro et al., 2014). Recent studies have demonstrated that RANKL promotes osteoclastogenesis by activating autophagy through the phosphorylation of b-cell lymphoma-2 (BCL2) at the S70 site in OC precursor cells (Ke et al., 2022). Mechanistically, the TRAF6-mediated ubiquitination of Beclin1 at the K117 locus is a prerequisite for RANKL-induced OC differentiation (Arai et al., 2019). Activation of extracellular signal-regulated kinase (ERK) and AKT by M-CSF in bone marrow macrophages is essential for macrophage proliferation and OC survival (Boyle et al., 2003). Environmental factors such as hypoxia and stress have been shown to promote autophagy in OCs. Specifically, the production of hypoxia-inducible factor 1-alpha upregulates BNIP3, which stimulates the expression of Beclin-1, ATG5, and LC3. This cascade subsequently enhances osteoclastogenesis through the upregulation of cathepsin K, T-cell nuclear factor (NFAT) c1, and matrix metalloproteinase 9 (Zhao et al., 2012). Zhong et al. induced differentiation and autophagy in RAW264.7 cells knocked out of Beclin1 gene by stimulating exogenous RANKL with interleukin-17 (IL-17). Their findings indicated that the repression of Beclin1 resulted in a diminution of the augmented effects of IL-17 on bone destruction and autophagy in RAW264.7 cells. Concurrently, the TAB3/ERK pathway was found to be blocked, thereby substantiating the involvement of the Beclin1/TAB3/ERK pathway in OCs autophagy. (Zhong, 2022). Elevated levels of Beclin-1 during OC differentiation have been reported, and mice lacking Beclin-1 in cells that express cathepsin K showed increased cortical bone thickness due to attenuated OC activity. Conversely, overexpression of Beclin-1 in cells resulted in enhanced autophagy-induced osteoclastogenesis and increased bone resorption *in vitro* (Arai et al., 2019). Autophagy also plays a critical role in OC migration and function. OC migration requires the rapid assembly and disassembly of podocyte rings formed by actin filaments. Kindlin-3, an essential adaptor protein in pedunculated nucleosomes, has been demonstrated to interact with LC3B, facilitating autophagy-mediated protein degradation. Inhibition of autophagy increases kindlin-3 levels, which enhances its interaction with integrin β3, consequently impeding the migratory capacity of OC. LC3 is identified as a critical target for OC migration (Zhang et al., 2020). Finally, OCs resorb bone through the ruffled border, formed by the fusion of secreted lysosomes with the bone-adherent plasma membrane. Rab7, a critical component of OC functionality, specifically localizes to the ruffled border in a process that is dependent on ATG5. ATG5 is crucial for binding LC3-Ⅱ to the ruffled border, and attenuation of ATG5 expression results in a decline in LC3-Ⅱ levels, which leads to a reduction in Cdc42 activity and the inhibition of OC function (Chung, 2012; DeSelm, 2011). Montaseri et al. proposed that autophagy exhibits a dual role in the regulation of OCs, and that when autophagy defects and oxidative stress are increased, OCs manifests in a pathological manner, ultimately leading to OP (Montaseri et al., 2020). Therefore, it is imperative to maintain a balanced level of autophagy, as it plays a pivotal role in the treatment of OP (Figure 3).

## 5 Regulation of autophagy by natural products

**FIGURE 4:**
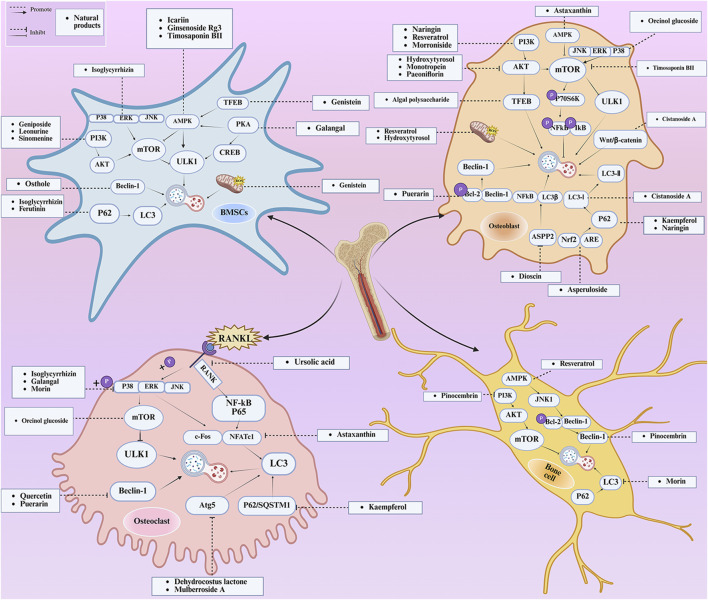
Mechanisms of autophagy and natural product regulation in OP. The natural products can activate various intracellular signaling pathways, induce cellular autophagy, promote the activity and bone formation of BMSCs, OBs, and bone cells, inhibit the activity and bone resorption of OCs, and suppress the occurrence of OP. The potential of natural products in combating OP is evidenced by their ability to modulate autophagy. P38, P38 mitogen-activated protein kinase; ERK,extracellular signal-regulated kinase; JNK, c-Jun N-terminal kinase; PI3K, phosphatidylinositol 3-kinase; AKT, protein kinase B; AMPK, AMP-activated protein kinase; PKA, protein kinase A; CREB, cyclic adenosine monophosphate response element-binding protein; ROS, reactive oxygen species; P70S6K, protein 70 S6 kinase; NFkB, nuclear factor kappa-light-chain-enhancer of activated B cells; IkB, inhibitor of NF-κB; Wnt/βcatenin, Wnt/β-catenin pathway; Nrf2, NF-E2-related factor 2; ARE, antioxidant response element; ASPP2, apoptosis-stimulating protein of p53-2; RANK, receptor activator of nuclear factor kappa B; RANKL, receptor activator of nuclear factor kappa B ligand; P65, a protein subunit of NF-κB; P62/SQSTM1, Sequestosome 1; c-Fos, c-fos protein; NFATc1, nuclear factor of activated T-cells 1.

A wide range of natural products have been identified as potential therapeutic agents for OP, particularly due to their role in promoting cellular autophagy. They play a therapeutic role in OP by promoting autophagy, facilitating the differentiation of BMSCs and mineralization of OBs, and promoting bone formation; or by inhibiting autophagy, thereby hindering the differentiation of OCs and reducing bone resorption; or by up-regulating the level of protective autophagy to prevent OC apoptosis under stress. The types of natural products and their mechanisms of action are summarized below (Table 1; Figure 4):

**TABLE 1:** Regulation of autophagy by natural products. OVX rats, osteoporotic changes in ovariectomized rats; rBMSCs, Rat bone marrow-derived mesenchymal stem cells; OVX mice, osteoporotic changes in ovariectomized rats; BMMs, Primary mouse bone marrow macrophages; MC3T3-E1 cells, mouse pre-osteoblastic cell line; RAW 264.7, murine macrophage cells; GIOP rats, Glucocorticoid-Induced Osteoporotic Rats; MLO-Y4 cells, murine long bone osteocyte Y4 cells; DPSC, dental pulp-derived stem cell; GK rats, Goto-Kakizaki rats; SAMP6, Characterization of senescence-accelerated mouse prone 6; BDL rats, bile duct-ligated rats.

Classifications	Natural products	Structural formula	Molecular formula	Primary sources	Animal model/cell model used in the study	Activation/inhibition of autophagy	Modes of action associated with autophagy	Therapeutic effects	References
Flavonoid	Genistein	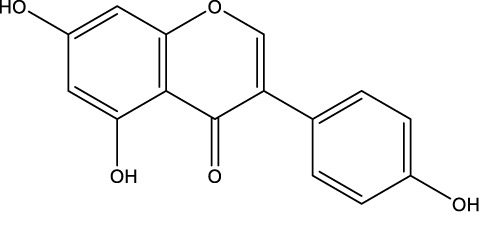	C_15_H_10_O_5_	Soybean products	OVX rats/rBMSCs	Activation	Upregulates active-β-catenin, SIRT3 expression	Activation of AMPK-ULK1 axis-mediated autophagy in adenomatous polyposis coli via TFEB upregulates the level of active-β-catenin protein, which in turn promotes osteogenic differentiation of BMSCs. In addition genistein agonizes ERRα, upregulates SIRT3 expression, and causes mitochondria to recruit Parkin, which in turn ubiquitinates the outer mitochondrial membrane, thus serving to protect BMSCs in OVX mice	Ge et al. (2023), Li et al. (2023b)
	Isoglycyrrhizin	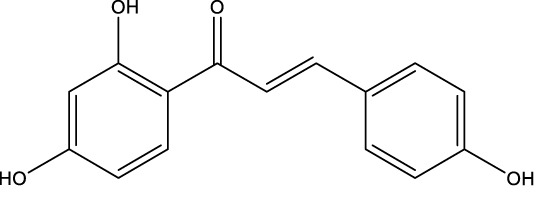	C_15_H_12_O_4_	licorice	OVX mice/rBMSCs	Activation	Increased LC3 levels and decreased p62 levels	Isoglycyrrhizin increases the level of LC3, an autophagy marker, and decreases the protein level of its autophagy substrate p62 in BMSCs, which improves the autophagic activity of BMSCs, and is able to activate the MAKP pathway to promote the osteogenic differentiation of BMSCs through p38/ERK autophagy	Su et al. (2024)
Flavonoid	Alpinetin	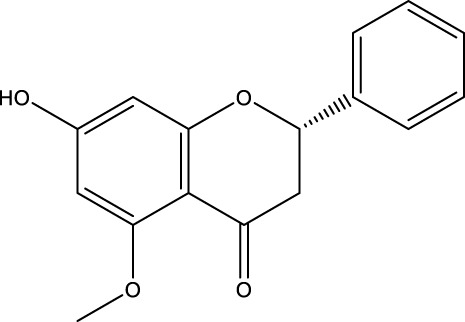	C_16_H_14_O_4_	Nutmeg and many other plants	BMSCs	Activation	Activates PKA, inhibits mTOR, and promotes ULK1 dephosphorylation	Alpinetin activates PKA, affects upstream mTOR/ULK1 signaling, and ultimately enhances the autophagy level of BMSCs and enhances their osteogenic differentiation by regulating PKA/mTOR/ULK1 autophagy pathway	Zeng et al. (2023a)
	Naringin	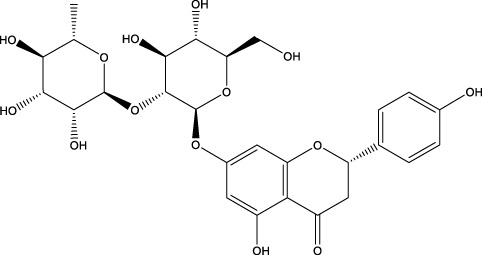	C_27_H_32_O_14_	Citrus fruits such as grapefruit and Chinese herbs	GIOP rats	Activation	Increased Beclin-1, p-AKT and p-mTOR expression and decreased p62 expression	Naringin increases Beclin-1 expression and decreases p62 expression in osteoblasts, while increasing AKT and mTOR phosphorylation levels and exerting a protective effect on glucocorticoid-induced OP through the PI3K/AKT/mTOR pathway	Ge and Zhou (2021)
Flavonoid	Quercetin	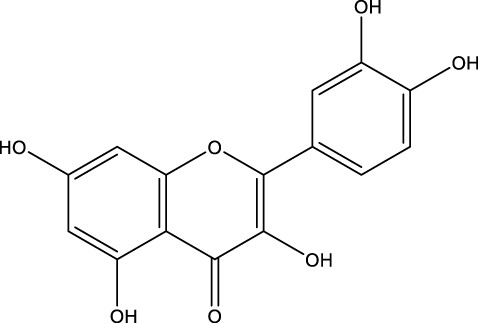	C_15_H_10_O_7_	Fruits, vegetables, tea	OVX rats	Inhibition	Reduced expression of PINP and Beclin1	Reduces the expression levels of PINP and Beclin1 in osteoblasts and prevents bone loss by inhibiting autophagy in osteoblasts	Xiong et al. (2023)
	Morin	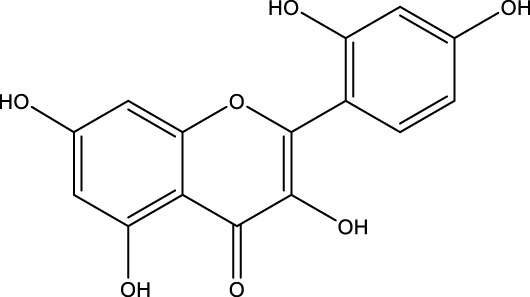	C_15_H_10_O_7_	Moraceae	OVX mice	Inhibition	Reduced LC3 and BECN1 protein levels	Morin reduces LC3 and BECN1 protein levels in osteoblasts of OVX mice and inhibits programmed death due to autophagy in osteoblasts after ovariectomy	Shi et al. (2021), Jiang et al. (2024)
	Pinocembrin	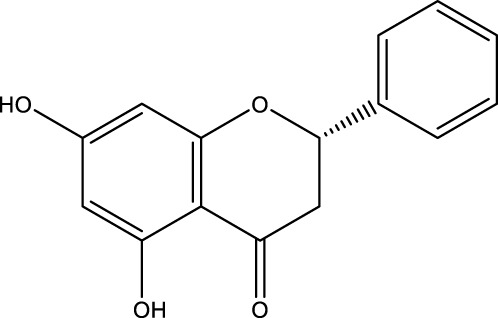	C_15_H_12_O_4_	Honey/Propolis	MLO-Y4 cells	Activation	Increased Beclin-1 and LC3B-II levels and decreased p62 levels	Pinocembrin increases Beclin-1 and LC3B-II levels, decreases p62 expression, and attenuates glucocorticoid-induced apoptosis of osteoblasts by inhibiting autophagy activation via PI3K/Akt/mTOR pathway in mouse long bone osteoblasts Y4 (MLO-Y4)	Wang et al. (2020)
	Icariin	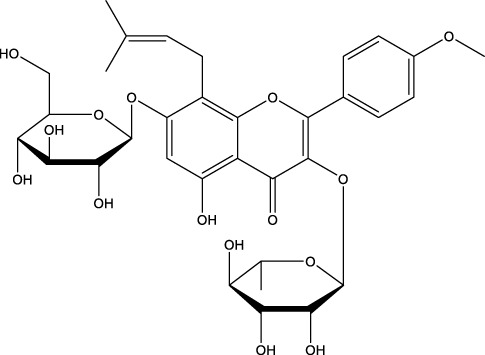	C_33_H_40_O_15_	Epimedium, genus of herbaceous flowering plants	OVX rats/rBMSCs	Activation	Upregulates ALP, mTOR, ULK1 Ser757, inhibits p-AMPK, p- ULK1 Ser555 PPAR-γ, p-mTOR, p62	Increasing autophagy levels, promoting osteogenic differentiation and mineralization, and inhibiting lipogenic differentiation in BMSCs	Liu, 2024; Zou et al. (2024)
Flavonoid	Galangin	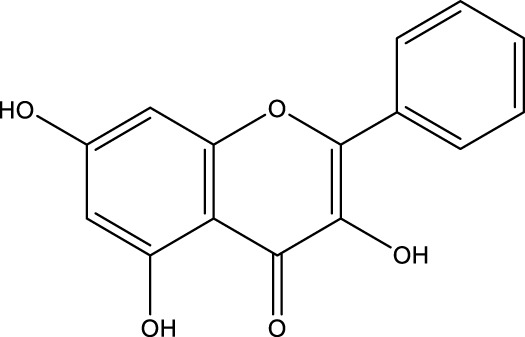	C_15_H_10_O_5_	Galangal Root	BMMs	Activation	Increased levels of Beclin-1, LC3B, pCREB, and p62/SQSTM1	Galangin increases the levels of beclin-1, LC3B, pCREB, and and p62/SQSTM1 in BMSCs from glucocorticoid-induced osteoporotic mice, and increases the level of PKA/CREB pathway-induced autophagy, which in turn promotes osteogenic differentiation of BMSCs	Li et al. (2021), Zeng et al. (2023b)
	Kaempferol	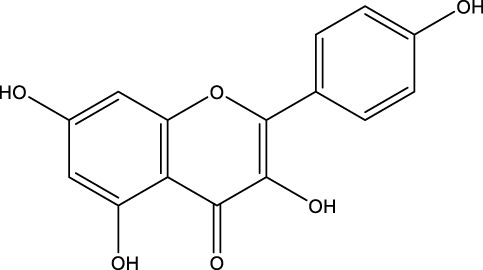	C_15_H_10_O_6_	Crabapple and many other plants	MC3T3-E1 cells/RAW 264.7	Activation·or Inhibition	Increased Beclin-1, SQSTM1/p62, LC3-II/LC3-I levels and decreased p62/SQSTM1 levels	Kaempferol can increase the expression of beclin-1 and SQSTM1/p62 in osteoblasts, promote the conversion of LC3-I to LC3-II, induce autophagy in osteoblasts in a dose-dependent manner, and promote osteogenic differentiation of osteoblasts. And kaempferol can strongly inhibit the expression of p62/SQSTM1 in osteoblasts, inhibit osteoblast autophagy, and induce cell apoptosis	Kim et al. (2016), Kim et al. (2018)
Flavonoid	Puerarin	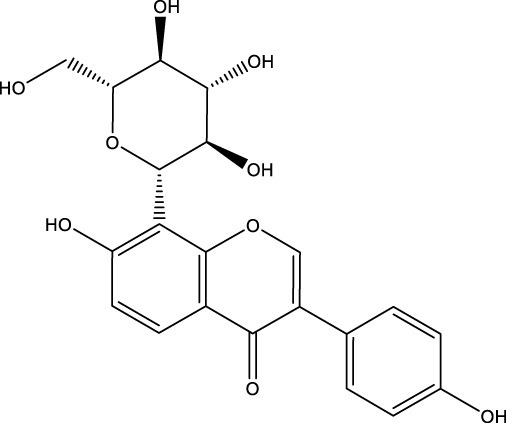	C_21_H_20_O_9_	Pueraria	BMMs/MC3T3-E1 cells	Activation or Inhibition	Decreased Beclin-1 expression in osteoclast precursors and increased p-Bcl-2 expression in osteoblast precursors	Puerarin was able to significantly inhibit the expression of Beclin-1 in osteoclast precursors, reduce the autophagy and proliferation levels of osteoclast precursors, and inhibit osteoclast differentiation. In addition, Puerarin was able to promote the phosphorylation of Bcl-2 at Ser70 site, the dissociation of Bcl-2- beclin1 complex, the entry of Beclin1 into autophagic flux, the enhancement of autophagy in osteoclast precursors, and the resistance to apoptosis in osteoclast precursors	Zhang et al. (2019), LI et al. (2024)
Polyphenol compound	Resveratrol	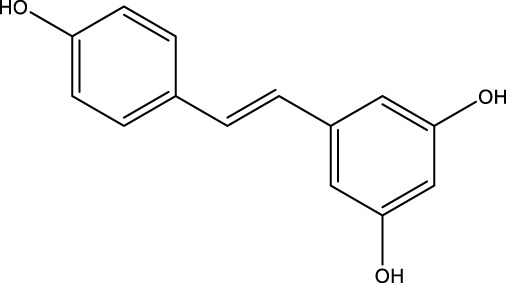	C_14_H_12_O_3_	Grape Leaves and Skins	MLO-Y4cells/MC3T3-E1 cells	Activation	Increased LC3 and Beclin-1 protein expression and downregulated p-AKT and p-mTOR	RES can increase LC3 and Beclin-1 protein expression, downregulate p-AKT and p-mTOR expression, and promote osteoblast autophagy by mediating the PI3K/Akt/mTOR pathway and increasing osteoblast SIRT1 expression. Restores osteoblast autophagy in the face of oxidative stress by activating AMPK/JNK1, which separates Bcl-2 and Beclin-1	Yang, 2019; Wei (2023), Cai et al. (2023), Ma et al. (2018)
Polyphenol compound	Hydroxytyrosol	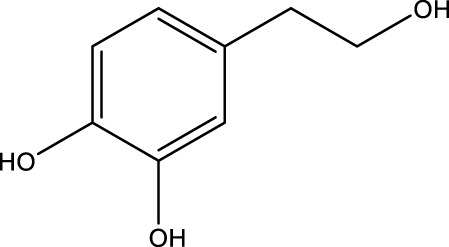	C_8_H_10_O_3_	Olive oil	N/A	Activation	Increased Beclin1 expression and inhibited mTOR expression	Hydroxytyrosol upregulates the AMPK signaling pathway, activates SIRT1, and downregulates the expression of the AKT/mTOR pathway to promote cellular autophagy, and is thought to promote osteoblast proliferation and inhibit osteoclast formation, increasing bone formation	De Pablos et al. (2019)
Terpenoid	Asperuloside	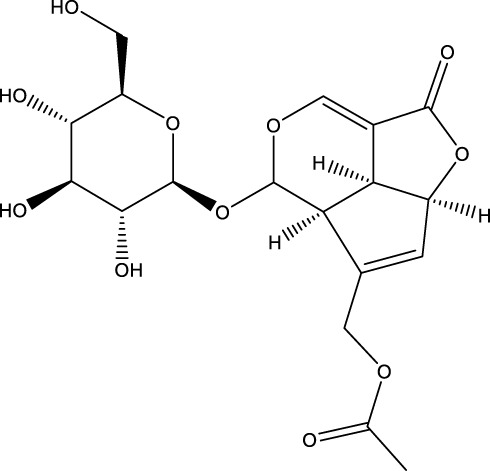	C_18_H_22_O_11_	Eucommiaceae, Rubiaceae, Gentianaceae	OVX rats/C57BL/6 rats/MC3T3-E1 cells	Activation	Upregulation of Beclin1, LC3II/I, Nrf-2 nuclear expression with p-AMPK/AMPK, inhibition of p62 phosphorylation with p-mTOR/mTOR expression	ASP can promote the nuclear expression of cellular Nrf-2 by activating the Nrf2-ARE pathway activity, inhibiting p62 phosphorylation, and up-regulating the expression of Beclin1 and LC3II/I, which promotes autophagy in osteoblasts, and thus increases osteoblast differentiation and mineralization. In addition, ASP not only increased the expression of Beclin1 and LC3II/I, but also increased the expression of p-AMPK/AMPK and inhibited the expression of p-mTOR/mTOR, which in turn activated the AMPK/mTOR pathway-mediated autophagy	Huang et al. (2024)
Terpenoid	Ferutinin	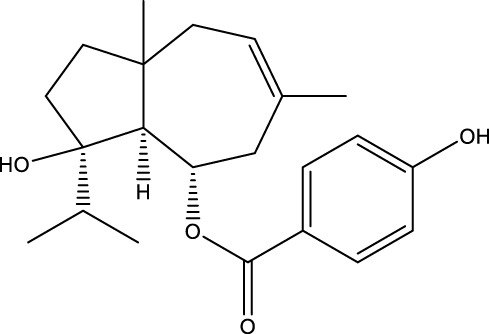	C_22_H_30_O_4_	Ferula	DPSCs	Activation	Upregulation of LC3B-II, BECN1, ATG3, ATG5 and ATG7 expression and downregulation of mTOR and p62 expression	Ferutinin induces KLF2-mediated autophagy in dental pulp stem cells, upregulates LC3B-II, BECN1, ATG3, ATG5 and ATG7 expression, and downregulates mTOR and p62, and promotes osteogenic differentiation of dental pulp stem cells	Maity et al. (2022)
	Monotropein	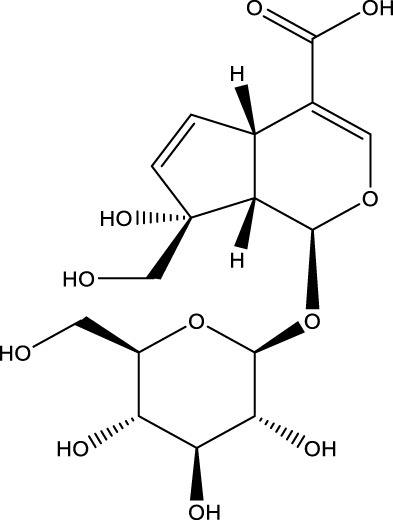	C_16_H_22_O_11_	*Morinda citrifolia* root	OBs	Activation	Enhancement of Beclin1 and LC3-II/LC3-I, inhibition of mTOR, p70S6K and 4EBP1	Monotropein increases Beclin1 expression and LC3-II/LC3-I ratio in osteoblasts, inhibits the phosphorylation of mTOR and its two downstream proteins (p70S6K and 4EBP1) as well as Akt, and increases the level of autophagy of osteoblasts via the Akt/mTOR pathway, protecting osteoblasts in oxidative stress environments	Shi et al. (2020)
Terpenoid	Geniposide	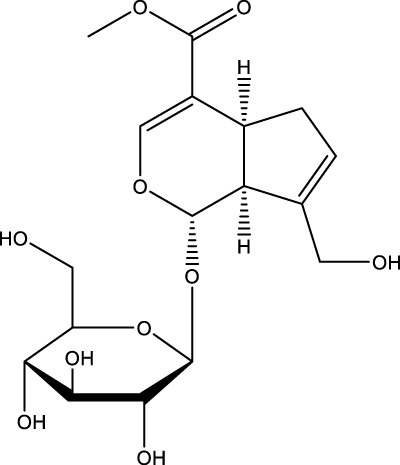	C_17_H_24_O_10_	Gardenia	MC3T3-E1 cells	Activation	Increased GLP-1R expression and decreased PI3K, AKT, mTOR expression	Geniposide activates the expression of GLP-1R, reduces the expression of PI3K/AKT/mTOR in MC3T3-E1 cells, and promotes autophagy through the GLP-1R/PI3K/Akt/mTOR pathway to facilitate their osteogenic differentiation	Huang et al. (2022)
	Dehydrocostus lactone	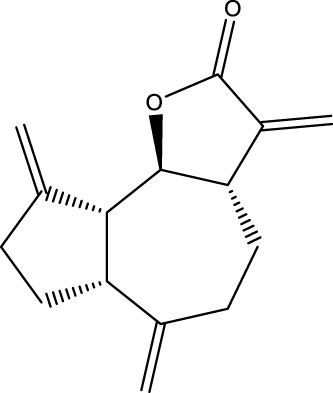	C_15_H_18_O_2_	Aucklandia lappa Decne	BMMs	Inhibition	Reduction of Atg5, cathepsin K, LC3-II/LC3-I	Dehydrocostus lacton significantly reduces the expression of Atg5 and cathepsin K and the conversion of LC3 in differentiated osteoblasts, decreases the secretion of mature cathepsin K protein, inhibits the expression of autophagy-related proteins, and inhibits the expansion and resorption activity of differentiated osteoblasts	Lee et al. (2019)
	Ursolic acid	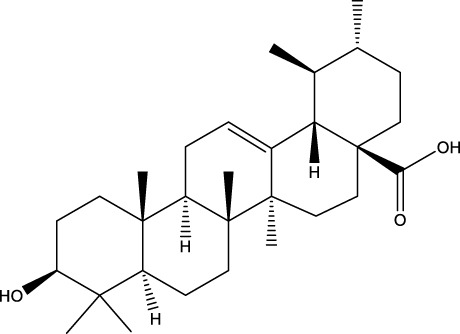	C_30_H_48_O_3_	Multifarious plants	OVX rats/MC3T3-E1 cells	Inhibition	Reduced expression of c-Fos, NFATc1, and LC3BII levels in osteoblasts	UA was able to reduce the expression of c-Fos and NFATc1 in osteoblasts and had a strong binding ability to LC3BII, interrupting RANKL-induced autophagy in osteoblasts by inhibiting the RANKL/NF-κB signaling pathway	Zheng et al. (2020), Lee et al. (2008)
Phytosaponins	Timosaponin BII	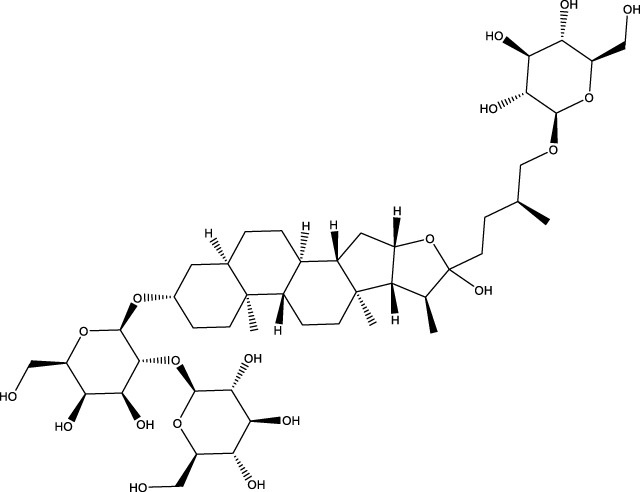	C_45_H_76_O_19_	Rhizomes of Zhi Mu	GK rats/OBs	Activation	Increased Beclin1, inhibited mTOR, S6K, p-NFκB, p-IκB expression	Timosaponin BII inhibits the phosphorylation levels of mTOR and S6K and their subordinate factors NFκB and IκB, upregulates Beclin1 expression, activates autophagy through inhibition of the mTOR/NFκB pathway, and attenuates high-glycemic environment-induced apoptosis in osteoblasts	Wang et al. (2021)
	Dioscin	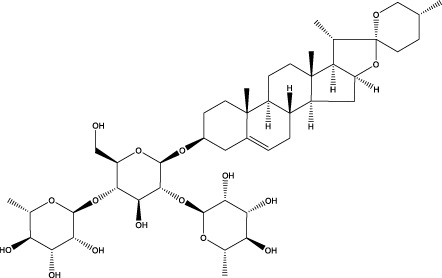	C_45_H_72_O_16_	Dioscorea opposita	MC3T3 - E1 cells	Inhibition	Increased ASPP2 expression and inhibited NF κβ, LC3β	Dioscin promotes the expression of ASPP2 and inhibits the expression of NF κβ and LC3β in MC3T3-E1 cells in a concentration-dependent manner, and promotes the proliferation and differentiation of osteoblasts by inhibiting autophagy mediated by the ASPP2/NF κβ pathway	Zhu et al. (2017)
Phytosaponins	Ginsenoside Rg3	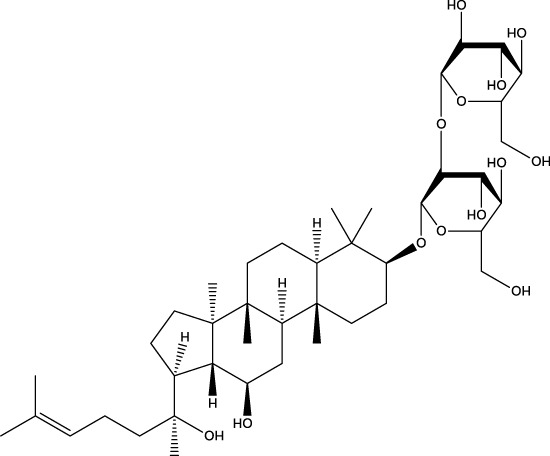	C_42_H_72_O_13_	Ginseng	OVX rast/MC3T3-E1 cells	Activation	Increases AMPK and inhibits mTOR	Ginsenoside Rg3 significantly enhances AMPK signaling and inhibits mTOR signaling in MC3T3-E1 cells, regulates autophagy through the per-AMPK/mTOR pathway, and promotes osteogenic differentiation and mineralization	Zhang et al. (2020)
Alkaloid	Tetramethylpyrazine	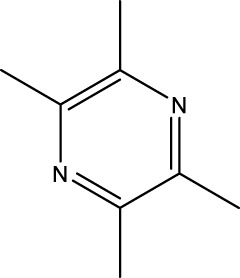	C_8_H_12_N_2_	Ligusticum chuanxiong	GIOP rats/rBMSCs	Activation	Increases AMPK and inhibits mTOR	TMP can organize excess glucocorticoid-induced apoptosis in BMSCs by activating the AMPK/mTOR pathway and increasing autophagy levels in BMSCs	Wang et al. (2017)
	Leonurine	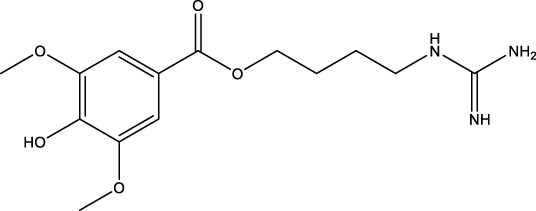	C_14_H_21_N_3_O_5_	Motherwort	rBMSCs	Activation	Increased Atg5, Atg7, LC3 mRNA and protein levels	Leonurine upregulates Atg5, Atg7 and LC3 mRNA and protein levels in mouse BMSCs, activates autophagy and promotes osteogenic differentiation of BMSCs through the PI3K/AKT/mTOR pathway	Zhao et al. (2021)
Alkaloid	Sinomenine	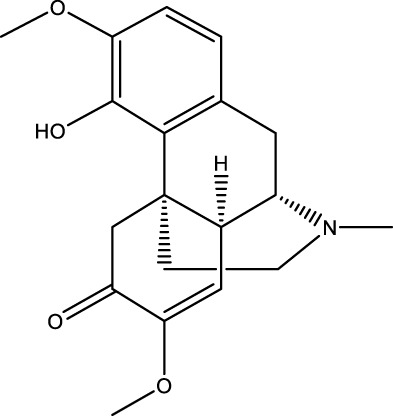	C_19_H_23_NO_4_	The root of Cymbopogon flexuosus	OVX mice/rBMSCs	Activation	Inhibition of AKT, mTOR	Sinomenine reduces the level of mTOR phosphorylation target protein AKT, inhibits mTOR activity, and activates autophagy and promotes osteogenic differentiation of BMSCs through the PI3K/AKT/mTOR signaling pathway in BMSCs	Xiao et al. (2024)
Carotenoids	Astaxanthin	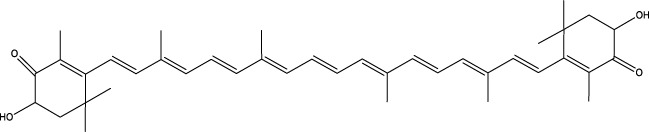	C_40_H_52_O_4_	Algae, plankton, crustaceans, fish, and bird feathers	BMCs	Activation	Improvement of Nrf2	Astaxanthin increases Nrf2 expression, activates the AMPK/mTOR pathway, and promotes osteoblast activity	Hwang et al. (2018), Alugoju et al. (2023), Balci Yuce et al. (2018), McCarty et al. (2022)
Phenylpropanoid	Cistanoside A	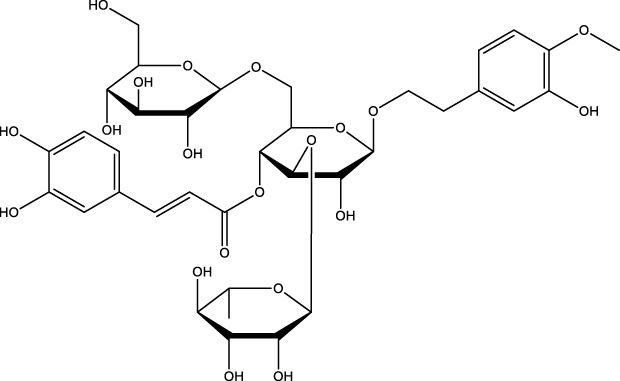	C_36_H_48_O_20_	Cistanche	OBs	Activation	Increased expression of LC3-I/II	Cistanoside A increases LC3-I/II expression, enhances Wnt/β-catenin signaling pathway activity, promotes autophagy in osteoblasts, and inhibits osteoblast apoptosis	Chen et al. (2022)
	Morroniside	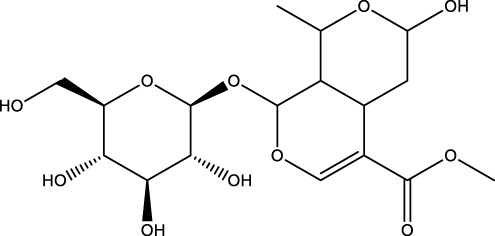	C_17_H_26_O_11_	Fruit of Cornus officinalis	MC3T3-EI cells	Activation	Enhancement of PI3K, Akt, and mTOR activities in MC3T3-EI and increase in Atg13 or Beclin1 protein levels	Morroniside increased the activities of PI3K, Akt and mTOR in MC3T3-EI, and promoted osteoblast differentiation through the PI3K/Akt/mTOR signaling pathway. When knocking down mTOR in MC3T3-EI enhanced morroniside upregulated autophagic activity and Atg13 or Beclin1 protein levels, and elevated Atg13 or Beclin1 protein promoted morroniside-regulated osteogenic differentiation	Liu et al. (2021), Li et al. (2022)
Phenylpropanoid	Paeoniflorin	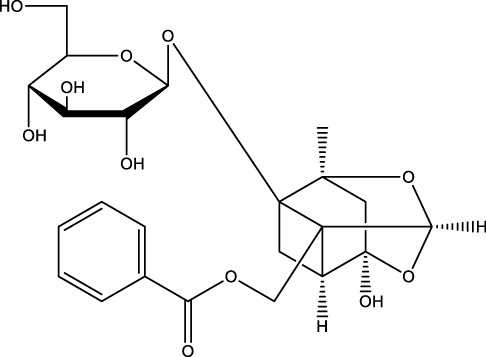	C_23_H_28_O_11_	Paeonia lactiflora	MC3T3-EI cells	Activation	Increased ALP activity, bone calcium and Beclin-1 expression and decreased CTX-1 levels	Paeoniflorin increases ALP activity with osteocalcin and Beclin-1 expression and decreases CTX-1 levels in dexamethasone-treated MC3T3-EI cells, increases autophagy levels in osteoblasts through inhibition of the AKT/mTOR signaling pathway, and thus promotes osteoblast differentiation and mineralization	Yang et al. (2021)
	Osthole	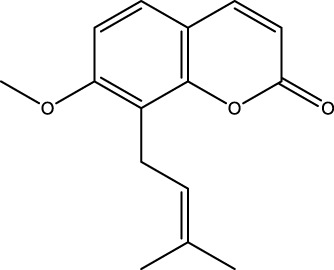	C_15_H_16_O_3_	Serpentine	rBMSCs	Activation	Increased protein and mRNA expression of Beclin1, LC3	Osthole was able to increase the protein and mRNA expression levels of Beclin1 and LC3, increase the autophagy level of BMMSC, and promote their differentiation to osteoblasts	Zheng et al. (2019)
Phenylpropanoid	Orcinol glucoside	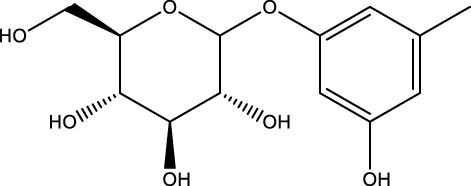	C_13_H_18_O_7_	Curculigo orchioides Gaertn	SAMP6/GIOP mice/OBs	Activation or Inhibition	Increased levels of mTOR phosphorylation in osteoclasts, phosphorylation of p38 in osteoblasts	Orcinol glucoside can inhibit osteoclastogenesis by activating the Nrf2/Keap1 pathway, increasing the levels of the antioxidant enzymes HO-1 and NQO-1, scavenging ROS, and inhibiting autophagy of osteoclasts by increasing the levels of mTOR phosphorylation in osteoclasts and activating the mTOR pathway.Orcinol glucoside can also directly bind to p38 protein and promote the phosphorylation of p38 to promote osteoclast activity	Gong et al. (2022), He et al. (2023)
	Mulberroside A	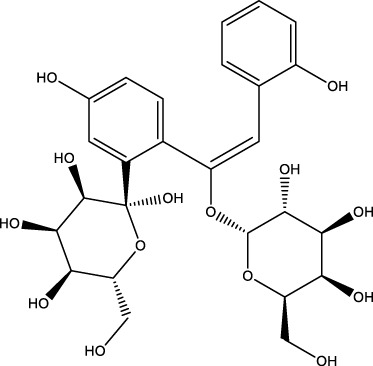	C_26_H_32_O_14_	Mulberry bark and branches	OVX mice/BMMs	Inhibition	Inhibition of Lc3, Atg16l1 and Atg5	Mulberroside A inhibits osteoclast differentiation and function by suppressing Mitf expression, leading to downregulation of Lc3, Atg16l1, and Atg5 expression, and does not affect osteogenesis	Xue et al. (2024)
Plant polysaccharides	Algal polysaccharide	N/A	N/A	Yeast and many other organisms	OVX rats/BDL rats/MC3T3-E1 cells	Activation	Increased Akt, TFEB expression levels, increased p-ERK levels	Algal polysaccharide can treat osteoporosis by inducing and enhancing Akt/TFEB pathway-dependent autophagic flow and alleviate osteoblast pyroptosis by promoting osteoblast autophagy. In addition, Algal polysaccharide can reduce osteoclast-mediated osteoclastogenesis by promoting autophagosome formation, increasing ERK phosphorylation in osteoblasts, and upregulating OPG secretion	Wang et al. (2023b), Xu et al. (2020)

### 5.1 Flavonoid

Flavonoid natural products have fundamental pharmacological effects, including antioxidant, anti-inflammatory, and anti-tumor properties (Soveral, 2013). Research has demonstrated that flavonoid natural products can enhance the activity and differentiation of OBs while inhibiting the activity of OCs by regulating autophagy.

A variety of flavonoid natural products have been demonstrated to activate autophagy pathways and differentiation signals in OBs and BMSCs, thereby significantly promoting bone formation. The key compounds include genistein, isoglycyrrhizin (ISL), alpinetin,and naringin. Genistein, from the legume family, is an isoflavonoid commonly found in soy products (Dixon and Ferreira, 2002). It has been demonstrated that genistein activates the AMPK-ULK1 axis-mediated autophagy in adenomatous polyposis coli via the transcription factor EB (TFEB). This activation results in the upregulation of the level of active-β-catenin protein, which in turn promotes the osteogenic differentiation of BMSCs in OVX rats. This process significantly inhibits bone loss and alleviates OP in OVX rats (Ge et al., 2023). Furthermore, genistein has been shown to play a protective role in BMSCs in OVX mice by agonizing estrogen-related receptor α (ERRα), upregulating SIRT3 expression, and causing mitochondrial recruitment of Parkin, which in turn ubiquitinates the outer mitochondrial membrane (Li M. et al., 2023). ISL is a flavonoid that is derived from licorice (Kobayashi et al., 1995)A growing body of research has demonstrated the potential of ISL in modulating autophagy in BMSCs. Studies have shown that ISL can enhance the expression of LC3, a marker of autophagy, and reduce the levels of p62, a substrate of autophagy. This modulation of autophagy has been found to increase the activity of BMSCs, leading to the activation of the MAKP pathway. This, in turn, has been observed to promote the osteogenic differentiation of BMSCs through p38/ERK autophagy. The results of these studies suggest that ISL can significantly enhance the formation of femur bone and reduce bone resorption in mice. This suggests that ISL may play a therapeutic role in the OP of OVX mice. The effect of BMSCs on OP in OVX mice was therapeutic (Su et al., 2024). Alpinetin is a naturally occurring flavonoid that is derived from nutmeg and a variety of other plants (Gul, 2022). It has been demonstrated that alpinetin possesses the capacity to activate protein kinase A (PKA), exert an influence on upstream mTOR/ULK1 signaling, and consequently augment the autophagy level of BMSCs by modulating the PKA/mTOR/ULK1 autophagy pathway. This process enhances their osteogenic differentiation and significantly mitigates dexamethasone (DEX)-induced bone loss in OP mice (Zeng et al., 2023a). Naringin, a naturally occurring citrus flavonoid, is predominantly derived from citrus fruits, such as grapefruit, and Chinese herbal medicine (Zhu et al., 2024). Recent findings have demonstrated that naringin has the capacity to enhance Beclin-1 expression and reduce p62 expression in OBs. In addition, it has been observed to elevate the phosphorylation levels of AKT and mTOR, thereby exerting a protective effect on glucocorticoid-induced OP through the PI3K/AKT/mTOR pathway (Ge and Zhou, 2021).

Quercetin (QR), morin, and pinocembrin have been demonstrated to impede autophagy-dependent bone destruction. QR is a species monomer belonging to the flavonoid subclass of Cortex Eucommiae, which is widely found in the daily intake of fruits, vegetables, tea, and other substances. (Deepika and Maurya, 2022). *In vitro* experiments demonstrated that QR promoted antioxidant hormone expression, reduced ROS production, and inhibited RANKL-induced differentiation of OCs in a dose-dependent manner (Tsuji et al., 2009; Niu et al., 2020). Xiong et al. observed the anti-osteoporosis effect of QR on a rat model of postmenopausal OP induced by bilateral ovariectomy (OVX) and found that the inhibitory effect of QR on OCs autophagy was comparable to that of CQ. The combination of QR and CQ has been shown to have a substantial impact on the expression of PINP and Beclin1, leading to a significant inhibition of autophagy in OCs. The efficacy of this combination is comparable to that of alendronate. (Xiong et al., 2023). Morin, a flavonoid derived from mulberry plant, has demonstrated significant potential in the treatment of various chronic diseases, including diabetes, arthritis, and heart disease (Caselli et al., 2016; Sinha et al., 2016). Morin has been demonstrated to inhibit the phosphorylation of ERK and p38 in OCs, which, in turn, has been shown to attenuate the expression of c-fos, reduce the nuclear translocation of NF-κB, and significantly alleviate OVX-induced OP in mice. This attenuation of OP is achieved by morin through the inhibition of NF-κB and MAPK pathways, thereby preventing the formation and function of Ocs (Shi et al., 2021). Morin has been demonstrated to reduce the levels of two proteins, LC3 and BECN1, in osteoblasts derived from OVX mice. In addition, it has been shown to inhibit the process of programmed death due to autophagy in osteoblasts following ovariectomy. Collectively, these findings suggest that morin may have potential as a therapeutic agent in the treatment of OP (Jiang et al., 2024). Pinocembrin is predominantly derived from honey and propolis (Wang H. et al., 2023). Recent findings have demonstrated that pinocembrin has the capacity to enhance Beclin-1 and LC3B-II levels while concomitantly reducing p62 expression in mouse long bone osteoblasts Y4. The observed effects of pinocembrin are attributed to its ability to stimulate autophagy, a process that is known to be suppressed by the PI3K/Akt/mTOR pathway. This stimulation of autophagy has been shown to attenuate glucocorticoid-induced apoptosis of osteoblasts and to alleviate glucocorticoid-induced OP (Wang et al., 2020).

It is noteworthy that Icariin (ICA), Galangin (GAL), kaempferol, and puerarin synergistically regulate the osteogenesis-osteoclast balance through multiple targets. These compounds promote the activation and differentiation of autophagy-dependent OBs and inhibit osteoclast activation, thereby achieving bidirectional optimization of bone homeostasis. ICA is a flavonoid that is extracted from Epimedium spp (He et al., 2020). The investigation revealed that ICA exhibited the capacity to impede OP of OVX rats by augmenting the autophagy level of BMSCs. This augmentation was concomitant with a promotion of their osteogenic differentiation and mineralization, alongside an inhibition of their lipogenic differentiation (Liang et al., 2019). In the treatment of OP, the administration of ICA at suitable concentrations has been demonstrated to promote bone formation and inhibit bone resorption through the BMP and Wnt/β-catenin signaling pathways, with no observed adverse effects (Zhang J. et al., 2010; Zhao and Shinkai, 2010). The ICA demonstrated notable anti-inflammatory effects on senescent macrophagesand was able to promote osteogenesis in BMSCs. This process was shown by transcriptomics to be regulated by the TNF-α signaling pathway, which is closely related to autophagy levels (Bai, 2023). Research has demonstrated that ICA possesses the capacity to regulate the expression of various enzymes and proteins involved in cellular processes. These include alkaline ALP, mTOR, and ULK1 Ser757, as well as p-AMPK and ULK1 Ser555 phosphorylation. The expression levels of peroxisome proliferator-activated receptor γ (PPAR-γ), phosphorylated mTOR (p-mTOR), and autophagy junction protein p62 (p62) suggest that ICA is involved in the activation and inhibition of AMPK/ULK1 and Akt/mTOR/ULK1 autophagy pathways in the treatment of OP (Liu, 2024; Zou et al., 2024). GAL is a flavonoid that is derived from the Galangal Root (Hassanein et al., 2023). Li et al. demonstrated that GAL impeded the nuclear translocation of p65 in RANKL and M-CSF-induced monocyte macrophages. Furthermore, GAL exhibited a dose-dependent inhibition of the phosphorylation of ERK and p38, suppression of the MAPK and NF-κB signaling pathways, and subsequent inhibition of the generation of OCs and lipopolysaccharide-induced bone resorption. This multifaceted action contributed to the therapeutic efficacy of OP (Li et al., 2021). Furthermore, it was demonstrated that GAL could enhance the levels of beclin-1, LC3B, pCREB, and p62/SQSTM1 in BMSCs from glucocorticoid-induced OP mice. Additionally, GAL increased the level of autophagy induced by the PKA/CREB pathway, thereby promoting osteogenic differentiation of BMSCs and alleviating glucocorticoid-induced OP (Zeng et al., 2023b). Kaempferol is a naturally occurring flavonoid that has been identified in a variety of plants, including those found in the genus Malus, more commonly known as the crabapple (Periferakis et al., 2022) The findings demonstrated that kaempferol, at concentrations below 10 μM, enhanced the expression of beclin-1 and SQSTM1/p62 in OBs, promoted the conversion of LC3-I to LC3-II, induced autophagy in OBs in a dose-dependent manner, and facilitated the osteogenic differentiation of OBs (Kim et al., 2016). It has been demonstrated that kaempferol can strongly inhibit the expression of p62/SQSTM1 in OCs, inhibit autophagy in OCs, induce apoptosis, and thereby play a role in the treatment of OP (Kim et al., 2018). Puerarin, a flavonoid extracted from the Pueraria, is a natural compound that has been studied for its potential health benefits (Zhou et al., 2014). Puerarin has been demonstrated to exert a substantial inhibitory effect on the expression of Beclin-1 in OCs precursors. This effect is accompanied by a reduction in the autophagy and proliferation levels of OCs precursors, as well as an inhibition of the differentiation of OCs. Consequently, these observations contribute to an alleviation of OP (Zhang et al., 2019). In addition, Puerarin was able to promote the phosphorylation of b-cell lymphoma-2 (Bcl-2) at the Ser70 site, the dissociation of the Bcl-2- beclin1 complex, the entry of Beclin1 into autophagic flux, the enhancement of autophagy in the precursor cells of OBs (MC3T3-E1), the resistance to apoptosis of MC3T3-E1, and the alleviation of OP (LI et al., 2024).

Flavonoid natural products have been demonstrated to modulate and reestablish equilibrium in bone metabolism through a process known as two-way autophagy. One side can activate autophagy in BMSCs or OBs through AMPK/mTOR, PKA/CREB, PI3K/AKT signaling pathways, thereby enhancing osteogenic differentiation and mineralization. Conversely, it has been observed that the administration of this substance can impede the process of autophagy in OCs, or alternatively, can stimulate excessive autophagy in OCs, thereby inducing their apoptosis and subsequent reduction in bone resorption. For instance, the combination of QR and CQ exhibited a substantially greater inhibitory effect on OCs compared to alendronate. Consequently, the multi-target regulation and synergistic pathway of Flavonoid natural products offer a novel approach for the development of highly effective and low-toxic OP treatment strategies.

### 5.2 Polyphenol compound

Polyphenols have been demonstrated to possess potent basic pharmacological effects, including antioxidant and anti-inflammatory properties (Bruno and Ghiadoni, 2018). These characteristics render them significant regulators of autophagy in OBs and Ocs.

The polyphenolic compounds under consideration, including resveratrol (RES) and hydroxytyrosol, have been demonstrated to offer protection to OBs and to impede the proliferation and differentiation of OCs. These compounds have been shown to not only promote bone formation but also to prevent bone destruction, thereby exerting a bidirectional regulatory effect on bone metabolism. RES, a polyphenolic phytoestrogen extracted from grape leaves and skins, is a prominent subject in research on polyphenols (Meyer et al., 2024). RES demonstrated the capacity to enhance SIRT1 expression in OBs in a dose-dependent and time-dependent manner. Furthermore, high doses of RES led to increased expression of LC3 and Beclin-1 proteins, downregulated p-AKT and p-mTOR expression, and increased OBs activity. Furthermore, RES led to a concurrent reduction in TOM20, Hsp60, p-Akt, and p-mTOR activities in DEX-treated OBs, with no significant impact on p-p38 and p-JNK activities. These observations suggest that RES can play a protective role for OBs by mediating the PI3K/Akt/mTOR pathway, increasing the expression of SIRT1 in OBs, and increasing bone density in OP rats (Yang, 2019). In an external setting, in the face of oxidative stress, RES mitigated OP in OVX-treated rats by activating AMPK/JNK1, separating Bcl-2 and Beclin-1, restoring bone cells autophagy, and attenuating apoptosis (Wei, 2023). It has been shown that RES promotes MC3T3-EI value-addition and the formation of calcium nodules and osteogenic differentiation of MC3T3-EI (Cai et al., 2023). The findings demonstrated that RES exhibited a substantial capacity to mitigate the inhibitory effect of lipopolysaccharide on OBs by enhancing mitochondrial function. Additionally, RES-loaded polylactic acid impeded the differentiation of OCs, thereby contributing to the alleviation of OP (Ma et al., 2018). Hydroxytyrosol is a constituent of olive oil (Bertelli et al., 2020). A growing body of research has demonstrated the potential of hydroxytyrosol to regulate the AMPK signaling pathway, activate SIRT1, and downregulate the expression of the AKT/mTOR pathway. This regulatory effect is believed to promote cellular autophagy, which in turn may stimulate the proliferation of OBs and inhibit the formation of OCs. Consequently, this regulatory effect could potentially enhance bone formation, suggesting a potential therapeutic application for OP (De Pablos et al., 2019).

Polyphenols have been shown to protect OBs and inhibit OCs by regulating key signaling pathways and autophagy. They have been shown to promote the proliferation, differentiation, and activity of OBs, while concurrently inhibiting the differentiation of OCs. Additionally, they have been observed to enhance mitochondrial function and reduce oxidative stress and inflammation-induced cell damage and apoptosis. Collectively, these effects promote bone formation and inhibit bone resorption, thereby contributing to the alleviation of OP.

### 5.3 Terpenoid

Terpenoids have fundamental pharmacological effects, including anti-inflammatory, antioxidant, and antibacterial properties (Atriya et al., 2023). Terpenoids have been demonstrated to exert a regulatory influence on the proliferation of OBs and the differentiation of OCs by modulating oxidative stress and autophagy.

A number of terpenoids, including asperuloside (ASP), ferutinin, monotropein, and geniposide, have been demonstrated to stimulate autophagy in both OBs and BMSCs. This process has been shown to offer several benefits, including the protection of OBs and BMSCs, the promotion of osteogenic differentiation, and a substantial enhancement of bone formation. ASP is a cyclic enol ether terpene belonging to a subclass of monoterpenes found primarily in plants from the Eucommiaceae and Rubiaceae families, as well as in select Gentianaceae plants (Chan et al., 2020). The study revealed that ASP could promote the nuclear expression of cellular Nrf-2 by activating the Nrf2-ARE pathway, inhibiting p62 phosphorylation, and up-regulating the expression of Beclin1 and LC3II/I. This, in turn, promoted autophagy in OBs, and consequently increased the differentiation and mineralization of OBs, and markedly attenuated OVX-induced OP (Huang et al., 2024). Ferutinin is a sesquiterpene ester that is primarily derived from Ferula (Safi et al., 2020). The findings indicated that ferutinin induced KLF2-mediated autophagy, elevated the expression of LC3B-II, BECN1, ATG3, ATG5, and ATG7, and reduced the expression of mTOR and p62 in pulp stem cells. This induction promoted the osteogenic differentiation of pulp stem cells, thereby alleviating OP (Maity et al., 2022). Monotropein, a naturally occurring iridoid glycoside derived from the root of *Morinda citrifolia*, is a monoterpene derivative with a variety of pharmacological effects, including cartilage protection and liver protection (Wang et al., 2014; Fang et al., 2023; Xie et al., 2024). Recent findings have demonstrated that monotropein exerts a positive effect on Beclin1 expression and the LC3-II/LC3-I ratio in OBs. In addition, monotropein has been shown to inhibit the phosphorylation of mTOR and its two downstream proteins (p70S6K and 4EBP1), as well as Akt. Furthermore, monotropein has been observed to increase the level of autophagy in OBs through the Akt/mTOR pathway. This protective effect of monotropein is particularly significant in environments subjected to oxidative stress, playing a crucial role in the prevention of OP (Shi et al., 2020). Geniposide is an iridoid glycoside that is predominantly derived from Gardenia (Ran et al., 2021). It has been demonstrated that Geniposide has the capacity to activate the expression of G protein-coupled receptor GLP-1R, reduce the expression of PI3K/AKT/mTOR in MC3T3-E1 cells, promote autophagy through the GLP-1R/PI3K/Akt/mTOR pathway, facilitate their osteogenic differentiation, and improve DEX-induced OP (Huang et al., 2022).

The presence of Dehydrocostus lactone, a terpenoid compound, was found to be effective in preventing bone destruction through the inhibition of autophagy in OCs. Dehydrocostus lactone, mainly derived from the traditional Chinese medicine Mucuna pruriens (Chen et al., 2020). Lee et al. demonstrated that the administration of dehydrocostus lactone led to a substantial reduction in the expression of ATG5 and cathepsin K, as well as the conversion of LC3, in differentiated OCs. This reduction was accompanied by a decrease in the secretion of mature cathepsin K protein, an inhibition in the expression of autophagy-related proteins, a suppression in the expansion and resorption activity of differentiated OCs, and a prevention of bone destruction in OVX mice (Lee et al., 2019).

Ursolic acid (UA), a terpenoid, has been shown to possess the capacity to impede the proliferation and differentiation of OCs while concurrently promoting the differentiation of OBs. This regulatory effect is achieved through the modulation of the autophagy pathway. Moreover, ursolic acid has been demonstrated to orchestrate bone homeostasis through a multifaceted collaborative regulatory network. UA is a pentacyclic triterpene that is derived from a variety of multifarious plants (Mlala et al., 2019). Zheng et al. demonstrated that UA can significantly reduce the expression of c-Fos and NFATc1 in OCs and has a strong binding ability with LC3BII, interrupting RANKL-induced autophagy in OCs by inhibiting the RANKL/NF-κB signaling pathway, thus inhibiting the proliferation and differentiation of OCs, and exerting a protective effect on OVX-induced OP in rats (Zheng et al., 2020). In addition, in the study conducted by Lee et al., has been shown that UA induces nuclear translocation of the p65 subunit of NF-kB in OBs, increases the expression levels of c-Fos and Fra-1, does not affect the expression of NFATc1 in OBs, and promotes the differentiation and mineralization of OBs through the activation of mitogen-activated protein kinase as well as transcription factors, such as NF-κB, to play a therapeutic role in OP (Lee et al., 2008).

In summary, terpenoids regulate the activities of both OBs and OCs in a dual manner. They achieve this by modulating oxidative stress, autophagy, and associated signaling pathways. As a result, terpenoids play a crucial role in the prevention and treatment of OP. For instance, UA and dehydrocostus lactone have been shown to target and inhibit the autophagy of OCs, thereby hindering the bone resorption activity of OCs. Conversely, ASP, Ferutinin, Monotropein, and Geniposide have been observed to promote autophagy of OBs and augment bone formation. Furthermore, the role of UA in promoting the differentiation and mineralization of OBs has been demonstrated.

### 5.4 Phytosaponins

Phytosaponins have been demonstrated to possess pharmacological effects, including anti-inflammatory, anti-oxidative, and analgesic properties (Passos et al., 2022). Phytosaponins have been demonstrated to elicit substantial therapeutic effects on OP by modulating autophagy in OBs.

The three phytosaponins examined in this study—namely, timosaponin BII, Dioscin, and ginsenoside Rg3—exhibited the capacity to promote autophagy in OBs, thereby enhancing bone formation. Timosaponin BII, the main active ingredient in the rhizomes of Zhi Mu, is a steroidal saponin (Xie et al., 2018). Recent findings have demonstrated the efficacy of Timosaponin BII in modulating mTOR and S6K phosphorylation, as well as their associated downstream factors NFκB and IκB. The study revealed that Timosaponin BII enhances Beclin1 expression, thereby activating autophagy. This process is facilitated by the suppression of the mTOR/NFκB pathway. The therapeutic potential of Timosaponin BII is further highlighted by its ability to mitigate apoptosis in the context of high-glucose environments in OBs. Additionally, it has been observed to contribute to the alleviation of deterioration in tibial microarchitecture and OP in diabetic rats (Wang et al., 2021). Dioscin is a natural steroidal saponin, mainly derived from Dioscorea spp (Yang et al., 2019). It has been demonstrated that Dioscin can promote the expression of apoptosis-stimulating protein p53-2 (ASPP2) and inhibit the expression of NFκβ and LC3β in MC3T3-E1 cells in a concentration-dependent manner. This, in turn, has been shown to promote the proliferation and differentiation of OB by inhibiting autophagy mediated by the ASPP2/NFκβ pathway. This process has the potential to prevent and treat OP (Zhu et al., 2017). Ginsenoside Rg3 is a promising active ingredient from the traditional Chinese medicine ginseng (Wu et al., 2024). It was shown that ginsenoside Rg3 significantly enhanced AMPK signaling and inhibited mTOR signaling in MC3T3-E1 cells, regulated autophagy through the per-AMPK/mTOR pathway, promoted osteogenic differentiation and mineralization, and had a therapeutic effect on OVX-induced OP (Zhang et al., 2022).

Phytosaponins (Timosaponin BII, Dioscin, Ginsenoside Rg3) are significant natural active substances that regulate autophagy of OBs and prevent and treat OP. They have the capacity to modulate autophagy in a variety of ways, including by interfering with core signaling nodes such as mTOR, AMPK, and NFκB. This has the potential to promote the function of OBs, increase bone formation, and enhance the therapeutic efficacy of OP.

### 5.5 Alkaloid

Alkaloids have been demonstrated to possess a variety of pharmacological effects, including anti-inflammatory, anti-oxidative, anti-cancer, and antibacterial properties (Thawabteh et al., 2019; Ti et al., 2021). Alkaloids have been demonstrated to enhance bone metabolism imbalance by modulating the autophagy pathway.

The three alkaloids identified in this study, including tetramethylpyrazine (TMP), leonurine, and sinomenine—have been shown to promote the osteogenic differentiation of BMSCs by increasing the level of autophagy. TMP is a significant alkaloid found in the traditional Chinese medicine Ligusticum chuanxiong (Lin et al., 2022). Wang et al. demonstrated that TMP could organize excess glucocorticoid-induced apoptosis of BMSCs and alleviate glucocorticoid-induced OP in rats by activating the AMPK/mTOR pathway and increasing the autophagy level of BMSCs (Wang et al., 2017). Leonurine, a principal active ingredient in traditional Chinese medicine derived from the motherwort, has been shown to possess significant biological activities (Liu et al., 2018). The findings indicated that 10 μM leonurine enhanced the expression of ATG5, ATG7, and LC3 mRNA and protein levels in mouse BMSCs. This treatment induced autophagy and promoted the osteogenic differentiation of BMSCs through the PI3K/AKT/mTOR pathway, which in turn prevented and treated OP (Zhao et al., 2021). Sinomenine is an alkaloid that is derived from the root of Cymbopogon flexuosus (Li J.-M. et al., 2023). XIAO et al. demonstrated that sinomenine effectively reduced the level of mTOR phosphorylation target protein AKT in BMSCs. In addition, sinomenine inhibited mTOR activity, activated autophagy in BMSCs through the PI3K/AKT/mTOR signaling pathway, promoted their osteogenic differentiation, and reduced bone loss in OVX-treated mice (Xiao et al., 2024).

Alkaloids, such as TMP, leonurine, and sinomenine, have been shown to enhance the autophagy level of BMSCs through their inherent anti-inflammatory and anti-oxidative properties. These alkaloids have been found to activate PI3K/AKT/mTOR and AMPK/mTOR signaling pathways. This approach has been demonstrated to effectively promote the differentiation of BMSCs into OBs, thereby enhancing the ability of bone formation and ultimately achieving the dual objectives of prevention and treatment of OP.

### 5.6 Plant polysaccharides

Plant polysaccharides are a category of biological macromolecules that have been demonstrated to possess immune regulatory, antitumor, anti-radiation, anti-inflammatory, anti-fatigue, and anti-aging properties (Huang et al., 2017).

Algal polysaccharide is a more stable natural disaccharide that has been found in various organisms, with a prevalence in yeast (Sharma et al., 2023). Studies have shown that algal polysaccharide, an mTOR-independent autophagy agonist, can treat OP by inducing and enhancing Akt/TFEB pathway-dependent autophagic flow (Wang Y. et al., 2023). Recent studies have demonstrated that algal polysaccharide alleviates OB pyroptosis by promoting OB autophagy. In addition, algal polysaccharide plays a role in the treatment of OP by promoting the formation of autophagosomes, increasing the phosphorylation of ERK in OBs, up-regulating the secretion of osteoprotegerin (OPG), and decreasing OB-mediated osteoclastogenesis (Xu et al., 2020).

Algae polysaccharide has been shown to protect OBs, promote their function, and inhibit the activity of OCs by activating MTOR-independent autophagy of OBs and enhancing ERK and OPG signals. This, in turn, regulates bone metabolism and improves OP. The distinctive MTOR-independent autophagy activation pathway of Algae polysaccharide constitutes a pivotal facet of its underlying mechanism of action.

### 5.7 Carotenoids

Carotenoids have been demonstrated to possess fundamental pharmacological effects, including antioxidant, anti-inflammatory, antineoplastic, and cardioprotective properties. Carotenoids have been demonstrated to promote bone metabolism by means of anti-oxidation and the regulation of autophagy (Milani et al., 2017).

Astaxanthin, a ketocarotenoid, is found in various sources, including certain types of algae, plankton, crustaceans, fish, and bird feathers (Lee et al., 2022). Research has demonstrated that astaxanthin possesses the capacity to impede the expression of nuclear factor c1 (NFATc1) in activated T cells. In addition, astaxanthin has been shown to hinder the RANK/RANKL signaling pathway, thereby reducing the production of OCs. This property of astaxanthin suggests a potential for utilization in the treatment of OP (Hwang et al., 2018; Alugoju et al., 2023). In addition, astaxanthin has been shown to increase the expression of Nrf2, activate the AMPK/mTOR pathway, promote the activity of OBs, and reduce alveolar bone loss in rats (Balci Yuce et al., 2018; McCarty et al., 2022).

Astaxanthin has been shown to inhibit OCs-mediated bone resorption by targeting RANKL/RANK/NFATc1. In addition, it has been demonstrated to enhance OBs function and antioxidant defense by activating Nrf2 and AMPK/mTOR. Consequently, these findings suggest that astaxanthin achieves bidirectional regulation of bone metabolism balance and is effective in the prevention and treatment of OP. This effect is indicative of the primary mechanism through which carotenoids enhance bone health by regulating autophagy.

### 5.8 Phenylpropanoid

Phenylpropanoids has been demonstrated to possess a variety of pharmacological effects, including anti-inflammatory, antioxidant, anti-cancer, and antibacterial properties (Carvalho et al., 2015; Álvarez-Martínez et al., 2021). Studies have indicated that it can enhance OP by modulating autophagy and functioning as an anti-inflammatory agent in bone metabolic cells.

Phenylpropanoids (Cistanoside A, morroniside, paeoniflorin, and osthole) have been demonstrated to significantly enhance osteogenic differentiation. The underlying mechanism of this effect involves the activation of the autophagy pathway and the modulation of key transcription factors. Cistanoside A, a phenylethanol glycoside derived from Cistanche, has been identified as a significant component of the plant’s composition (Xiong et al., 1996). The findings indicated that 10 µM Cistanoside A was capable of increasing the expression of LC3-I/II, enhancing the activity of the Wnt/β-catenin signaling pathway, promoting autophagy, inhibiting apoptosis of OBs, and increasing the differentiation of OBs. Consequently, these effects contributed to the alleviation of OP (Chen et al., 2022). Morroniside, a cyclic enol ether terpene glycoside found in the fruits of Cornus officinalis, is a promising candidate for further study (Yi et al., 2022). Morroniside has been demonstrated to exert a stimulatory effect on the activities of PI3K, Akt, and mTOR in MC3T3-EI. Furthermore, it has been shown to promote the differentiation of OBs through the PI3K/Akt/mTOR signaling pathway. In addition to these effects, morroniside has been shown to attenuate OP in OVX mice (Liu et al., 2021). Furthermore, the suppression of mTOR in MC3T3-EI enhances morroniside-induced autophagy activity and protein levels of ATG13 or Beclin1. Elevated protein levels of ATG13 or Beclin1 promote morroniside-regulated osteogenic differentiation and increase bone mass in OVX-treated mice (Li et al., 2022). Paeoniflorin, a chemical compound found in Paeonia lactiflora, is a well-documented active ingredient in traditional medicine (Zhang and Wei, 2020). It was shown that Paeoniflorin increased ALP activity with osteocalcin and beclin-1 expression, decreased the levels of type I collagen Bax and c-terminal terminal peptide in DEX-treated MC3T3-EI cells, increased the autophagy level of OBs by inhibiting the AKT/mTOR signaling pathway, which in turn promoted OBs differentiation and mineralization, and alleviated DEX induced OP in mice (Yang et al., 2021). Osthole, a coumarin analog in the phenylpropanoid class, is a constituent of the traditional Chinese medicine known as serpentine (Jarząb et al., 2017). The findings indicated that Osthole enhanced the protein and mRNA expression levels of Beclin1 and LC3, augmented the autophagy level of BMSCs, promoted its differentiation to OBs, and effectively alleviated OP caused by estrogen deficiency. However, the precise mechanism by which Osthole exerts these effects remains to be elucidated through further research (Zheng et al., 2019).

Two compounds in phenylpropanoids, Orcinol glucoside (OG) and Mulberroside A (Mul-A), have been demonstrated to offer protection to bone tissue by impeding autophagy-dependent activation of OCs. Mul-A’s effects on OCs differentiation and function are intriguing. Intriguingly, Mul-A did not affect osteogenesis. OG, a phenolic glucoside analog, is extracted from Curculigo orchioides Gaertn (Wu et al., 2005). GONG et al. demonstrated that OG could inhibit the generation of OCs by activating the Nrf2/Keap1 pathway, increasing the levels of the antioxidant enzymes HO-1 and NQO-1, scavenging ROS to inhibit the generation of OCs, and inhibiting the autophagy of OCs by increasing the levels of mTOR phosphorylation in OCs and activating the mTOR pathway. This alleviated OP in aged mice (Gong et al., 2022). In addition, OG binds directly to p38 protein, promotes p38 phosphorylation, enhances OB activity, and ameliorates glucocorticoid-induced OP in mice (He et al., 2023). Mul-A, a streptavidin compound derived from the bark and twigs of Morus alba, is a phenylpropanoid (Jo et al., 2014). Mul-A inhibits OC differentiation and function by suppressing the expression of microphthalmia-associated transcription factor, leading to downregulation of LC3, ATG16l1, and ATG5, without affecting osteogenesis (Xue et al., 2024).

The effects of phenylpropanoids on autophagy are highly context-dependent. For instance, Mul-A has been shown to impede the expression of MITF and to reduce the expression of autophagy-related genes, thereby hindering the autophagy of OCs and preventing excessive bone resorption. OG has been demonstrated to activate the Nrf2/Keap1 pathway, inhibit OCs autophagy, prevent excessive bone resorption, and enhance OBs activity by promoting p38 phosphorylation. Cistanoside A, morroniside, and paeoniflorin have been shown to activate autophagy in OBs through core autophagy and to involve Wnt/β-catenin, p38 MAPK, PI3K/Akt, and other key signaling pathways. Osthole has been demonstrated to activate autophagy in BMSCs, thereby promoting bone protection. The potent pharmacological effects of phenylpropanoids, including their anti-inflammatory and antioxidant properties, render them efficacious in combating a range of predisposing factors associated with OP.

## 6 The adverse effects of natural products

To date, no biologically active substance has been identified that is both pharmacologically active and entirely free from non-specific, off-target effects on normal tissues (Guo et al., 2023). When natural products are used in combination with drugs, precipitated natural products may alter the drug’s effects, either enhancing or reducing its activity, and potentially leading to toxicity (Gaston et al., 2020). High doses of quercetin have been reported to cause a 10% fluctuation in body weight in mice, and administration of 40,000 ppm quercetin resulted in renal tubular adenomas and adenocarcinomas in male mice, suggesting some carcinogenic activity in male F344/N rats (National Toxicology Program, 1992). Studies have shown that prolonged exposure to genistein can accelerate of abnormal estrous cycles in female Sprague-Dawley rats, with evidence of carcinogenic potential from chronical administration of genistein in these rats (Tr, 2024). Furthermore, high doses of RES have been found to adversely affect metabolic status, endothelial health, inflammation, and cardiovascular markers in humans (Ramírez-Garza et al., 2018). In addition, doses of 2.5g–5g of RES may cause mild to moderate gastrointestinal symptoms (Brown et al., 2010). Dose-limiting toxicities, such as hepatotoxicity and diarrhea, have been reported with intravenous infusions of 74, 98, and 130 mg/m2 of RES (Sun et al., 2020a).

In conclusion, the toxicity and safety of natural products must be carefully considered in clinical applications (Sun et al., 2020b). Currently, natural products have not been widely adopted in clinical practice, but they may possess inherent toxicity to normal tissues, including carcinogenicity and liver and kidney toxicity, while simultaneously exerting therapeutic effects on OP through multi-targets and methods. Furthermore, many natural products exhibit low oral bioavailability. Consequently, future research should focus on: (1) improving the safety profile of natural products by reducing toxicity and side effects, (2) optimizing drug administration methods, and (3) enhancing the bioavailability of these drugs (Davatgaran Taghipour et al., 2019).

## 7 Discussion and conclusion

OP is a major chronic disease affecting older adults, especially menopausal women, where the lack of estrogen due to menopause is the primary trigger for the disease (Srivastava and Deal, 2002; Yong and Logan, 2021). Research suggests that approximately 80–90 percent of adults in secondary prevention settings fail to receive appropriate management for OP (Brown, 2021). Bone is continuously undergoing a dynamic cycle of resorption and formation, a process that involves various bone cells such as BMSCs, OBs, and OCs. Abnormalities in the number or activity of these cells can disrupt bone formation and resorption, leading to bone diseases, especially OP. Fractures are the most common complication of OP and is the leading cause of morbidity and mortality in affected individuals. OP-related fractures significantly increase hospitalization and healthcare costs, reduce patients’ quality of life, overburden healthcare resources, and lead to significant personal and societal economic losses (Woodhead and Moss, 1998; Andersen, 2007). Thus, OP has become a significant public health problem.

A variety of pharmaceutical agents is currently available for the prevention and treatment of OP,. including recombinant parathyroid hormone, estrogen, bisphosphonates, and calcium. These drugs inhibit excessive bone resorption and promote bone formation by enhancing OB activity or inhibiting OC function (Rachner et al., 2011; Brown, 2022). However, the use of these agents is limited by their substantial expense and potential adverse effects, including cardiovascular complications, an increased risk of pathologic fractures, a higher likelihood of bone tumors, and immune system dysfunction (Khosla, 2009). These side effects have prompted researchers to study natural compounds more thoroughly. Natural products, which include various metabolites and chemical components derived from animal and plant extracts, insects, microorganisms, marine organisms, and endogenous chemicals present in human and animal systems, are known for their multi-component synergism, multi-target interactions, and diverse pathways. In comparison with traditional anti-OP drugs, natural products are safer, more convenient, and cost-effective. In addition, multi-targeted natural products overcome the shortcomings of single-target therapies in conventional anti-OP treatments. As a result, natural products have important medicinal value and great potential for market development. Research has also revealed novel mechanisms, including cellular autophagy, through which natural products may treat OP. In this article, we summarized the existing studies on how natural products could treat OP through the cellular autophagy pathway by searching and screening the literature. Autophagy is a cellular degradation process that plays an important role in maintaining cellular homeostasis and energy balance by transporting intracellular substances to lysosomes, where they are degraded and recirculated. Although autophagy is generally a protective process, excessive autophagy or disruption of its mechanism can lead to cell death (Liu et al., 2023). Thus, autophagy modulators may produce unexpected *in vivo* effects that could either benefit or harm the patient. When natural products are used as autophagy modulators for the treatment of OP, alterations in autophagy could have different effects on the endocrine system or other body systems. Despite their high potential, most studies on natural products for the treatment of OP are still limited to animal models, and there is a lack of clinical validation to assess their therapeutic effects on human. Furthermore, certain natural products exert a dual regulatory effect, simultaneously inhibiting bone resorption and promoting bone formation. However, many studies focus on single targets or pathways, failing to explore the interactions between these targets and pathways. Given the diverse sources and mechanisms of action of natural products, further research is required to develop safe and clinically effective treatments for OP.

In this review, we aimed to elucidate the relationship between cellular autophagy and OP. Autophagy plays an important role in bone remodeling in both physiological and pathological conditions. Additionally, we summarized and analyzed current natural products that modulate autophagy for the treatment OP, highlighting diverse strategies for managing the disease in OBs and OCs. However, further research is needed to identify suitable natural products, improve their bioavailability, reduce toxicity, and translate these findings into clinical applications. Future studies could focus on combining these natural products to develop new drug combinations or target specific autophagy-related mechanisms, offering a promising and potentially effective therapeutic strategy for the management of OP.
